# Supplementary material for: Multilingual translation for zero-shot biomedical classification using BioTranslator
Source: Nat Commun. 2023 Feb 10;14:738. doi: 10.1038/s41467-023-36476-2 (PMC9911740; doi:10.1038/s41467-023-36476-2)
Supplement: Supplementary file 1 — Supplementary Information [file 41467_2023_36476_MOESM1_ESM.pdf]

Supplementary Infomation

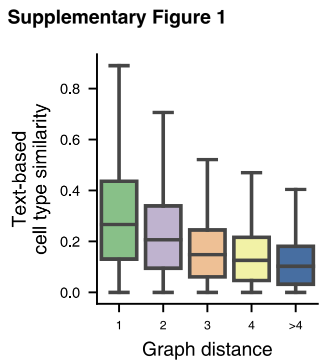

Supplementary Figure 1. Box plot comparing text-based cell type similarity with graph-based cell type distance on the Cell Ontology. For this box plot, the minima is the lower quartile (Q1) − 1.5*interquartile range (IQR), the maxima is the upper quartile (Q1) + 1.5*interquartile range (IQR), the centre is median (Q2), the bounds of box are Q1 and Q3, the whiskers are from the minima to Q1 and from Q3 to the maxima. n=3144, 17376, 64141, 178739, 2175336 cell type pairs examined when the graph-based cell type distance is 1, 2, 3, 4 and >4 respectively.

Supplementary Figure 2

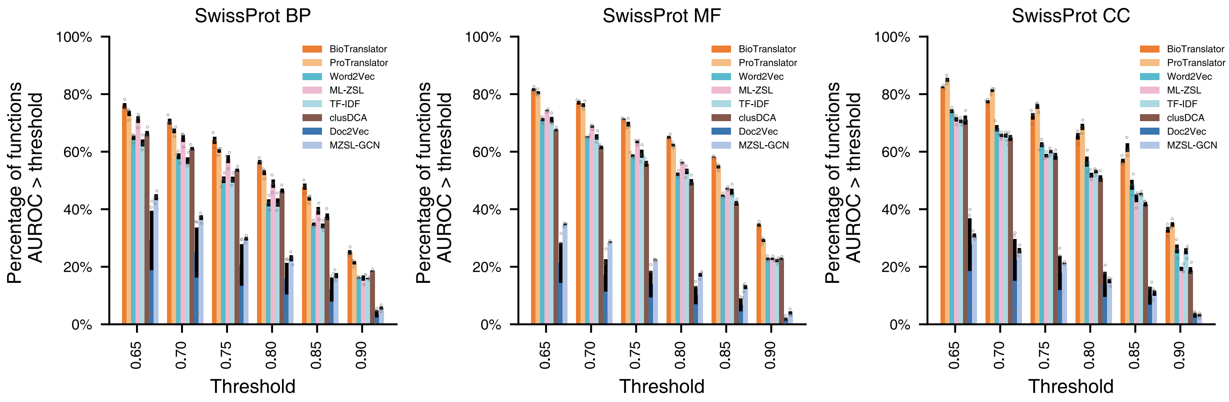

Supplementary Figure 2. Bar plot comparing the performance of BioTranslator to existing methods on SwissProt. y-axis is the percentage of GO terms with AUROC greater than different thresholds (x-axis). Error bar represents the standard errors in 3-fold cross-validation. Center of the error bar is the mean value.

Supplementary Figure 3

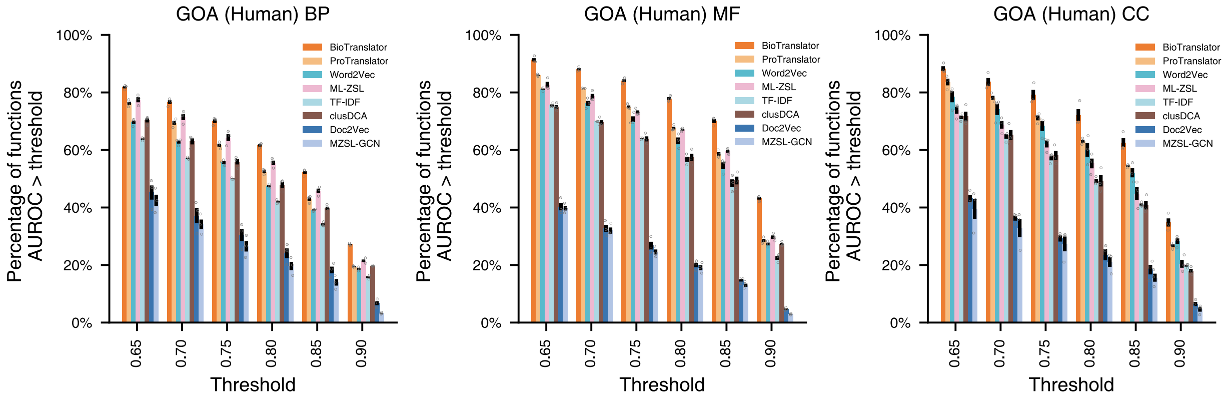

Supplementary Figure 3. Bar plot comparing the performance of BioTranslator to existing methods on GOA (Human). y-axis is the percentage of GO terms with AUROC greater than different thresholds (x-axis). Error bar represents the standard errors in 3-fold cross-validation. Center of the error bar is the mean value.

Supplementary Figure 4

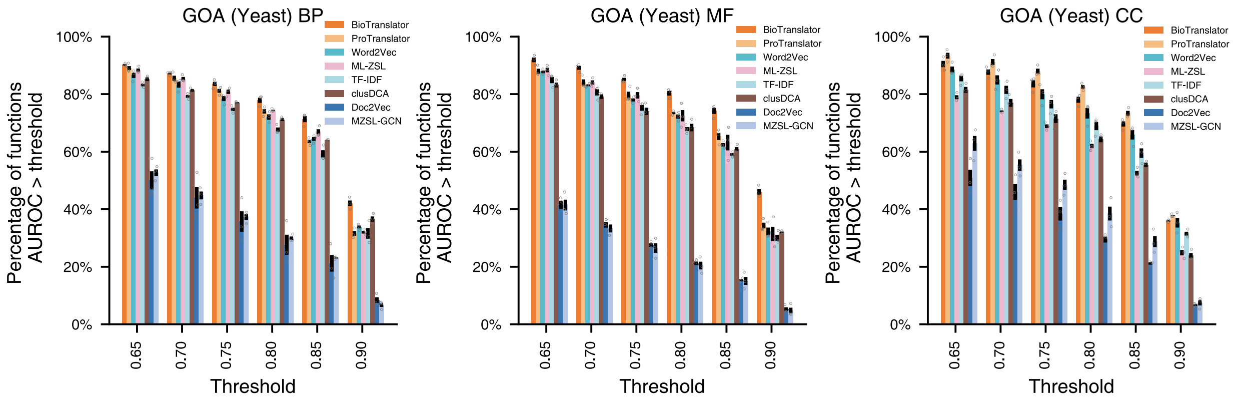

Supplementary Figure 4. Bar plot comparing the performance of BioTranslator to existing methods on GOA (Yeast). y-axis is the percentage of GO terms with AUROC greater than different thresholds (x-axis). Error bar represents the standard errors in 3-fold cross-validation. Center of the error bar is the mean value.

Supplementary Figure 5

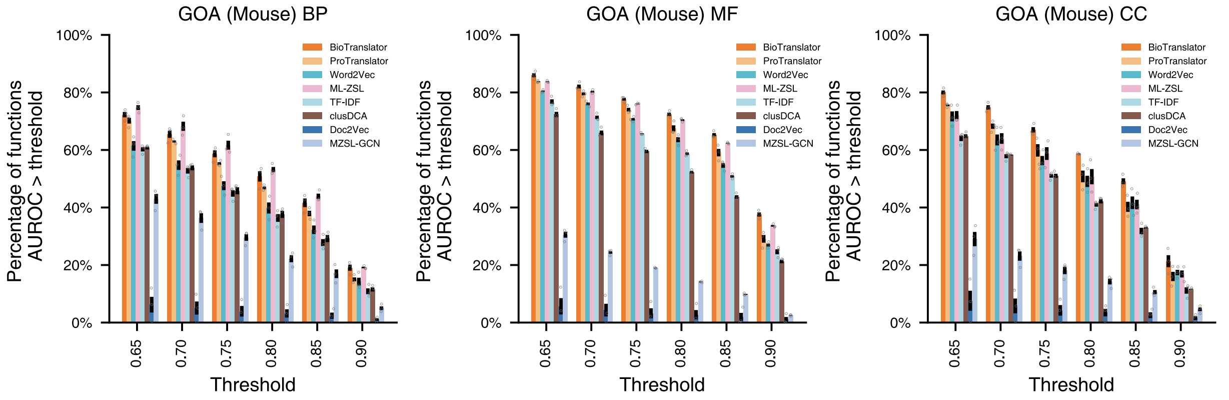

Supplementary Figure 5. Bar plot comparing the performance of BioTranslator to existing methods on GOA (Mouse). y-axis is the percentage of GO terms with AUROC greater than different thresholds (x-axis). Error bar represents the standard errors in 3-fold cross-validation. Center of the error bar is the mean value.

Supplementary Figure 6

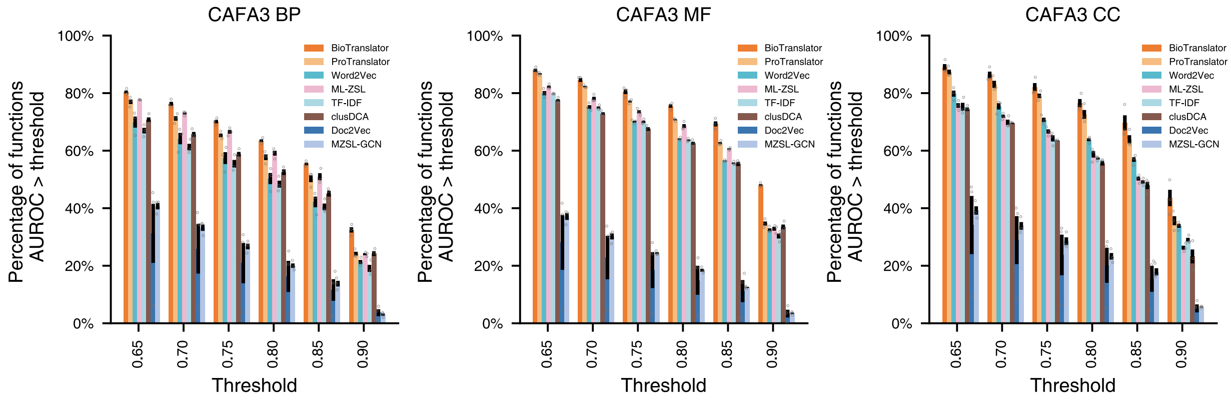

Supplementary Figure 6. Bar plot comparing the performance of BioTranslator to existing methods on CAFA3. y-axis is the percentage of GO terms with AUROC greater than different thresholds (x-axis). Error bar represents the standard errors in 3-fold cross-validation. Center of the error bar is the mean value.

Supplementary Figure 7

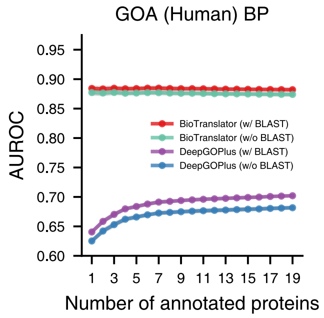

Supplementary Figure 7. Plot comparing the AUROC of using BioTranslator (w/ Blast), BioTranslator (w/o Blast), DeepGOPlus (w/ Blast) and DeepGOPlus (w/o Blast) for function annotation on GOA (Human) biological process domain. The protein annotation number of functions is from 1 to 20. The error bands represent the standard error across 3-fold cross-validation.

Supplementary Figure 8

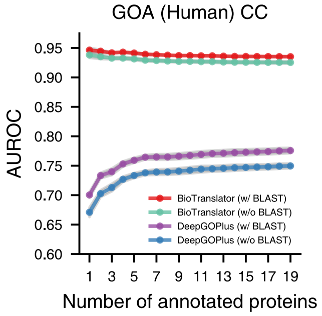

Supplementary Figure 8. Plot comparing the AUROC of using BioTranslator (w/ Blast), BioTranslator (w/o Blast), DeepGOPlus (w/ Blast) and DeepGOPlus (w/o Blast) for function annotation on GOA (Human) cellular component domain. The protein annotation number of functions is from 1 to 20. The error bands represent the standard error across 3-fold cross-validation.

Supplementary Figure 9

	phosphatidylglycerol metabolic process
Text generated by BioTranslator	The chemical reactions and pathways resulting in the formation of phosphatidyleth-anolamine, any of a class of glycerophospholipids in which a phosphatidyl group is esterified to the hydroxyl group of ethanolamine.
Text of the nearest term	Remodeling the acyl chains of phosphatidylglycerol, through sequential deacylation and re-acylation reactions, to generate phosphatidylglycerol containing different types of fatty acid acyl chains.
Ground truth text	The chemical reactions and pathways involving phosphatidylglycerols, any of a class of phospholipids in which the phosphatidyl group is esterified to the hydroxyl group of glycerol. They are important constituents of cell membranes.
	positive regulation of microtubule polymerization
Text generated by BioTranslator	Any process that activates or increases the frequency, rate or extent of microtubule polymerization or depolymerization.
Text of the nearest term	The addition of tubulin heterodimers to one or both ends of a microtubule.
Ground truth text	Any process that activates or increases the frequency, rate or extent of microtubule polymerization.

Supplementary Figure 9. Table showing the generated text, ground truth text and the text of the nearest term. The generated text, ground truth text and the text of the nearest term on the GO graph for GO term phosphatidylglycerol metabolic process and GO term positive regulation of microtubule polymerization pathway.

Supplementary Figure 10

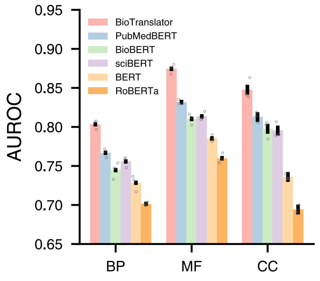

Supplementary Figure 10. Bar plot comparing the performance using different pretrained language models, including PubMedBERT, BioBERT, SciBERT, RoBERTa and BERT, on GOA (Human). BP, MF, CC strand for biological process, molecular function and cellular component. Error bar represents the standard errors in 3-fold cross-validation. Center of the error bar is the mean value.

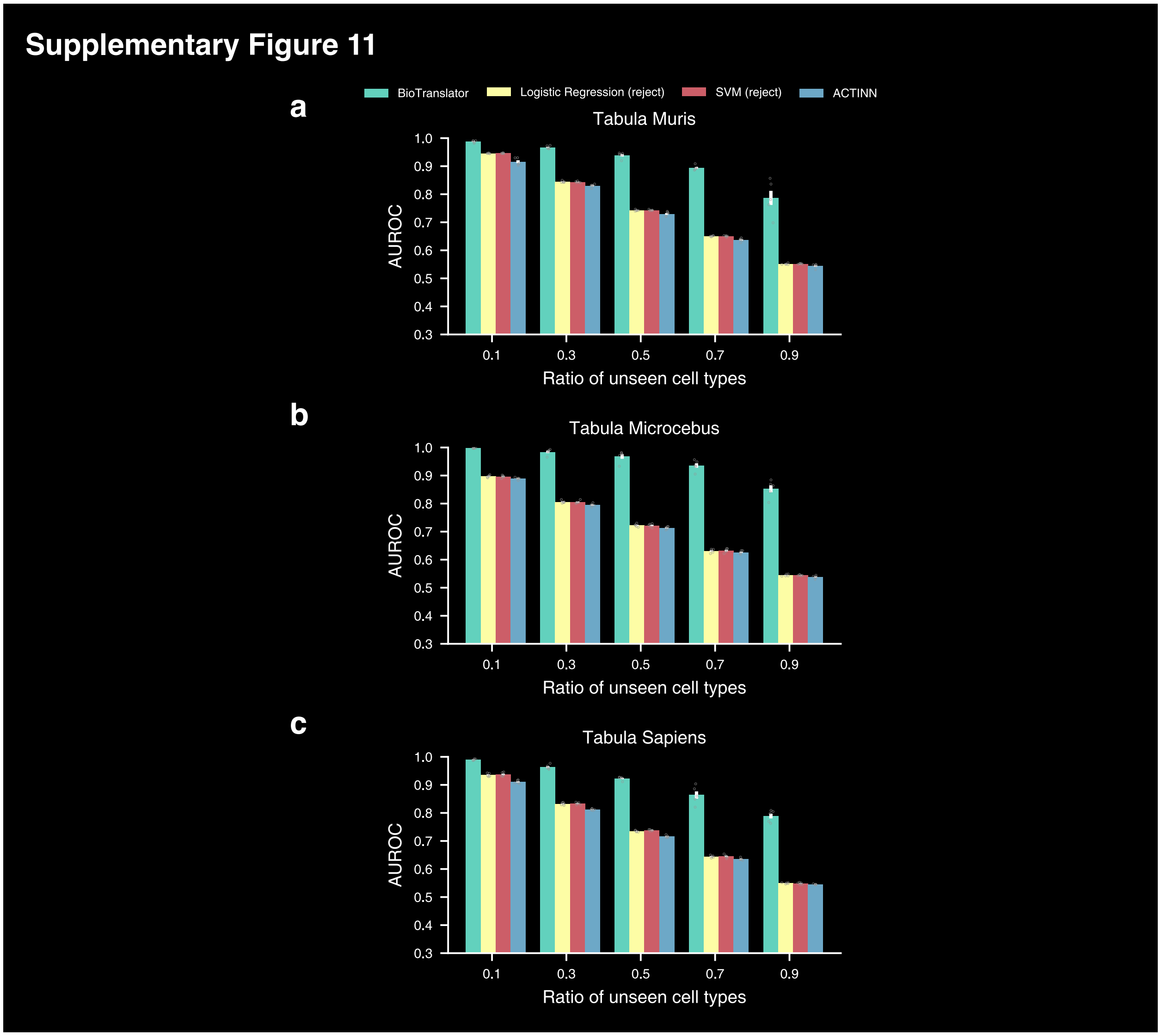

Supplementary Figure 11. a,b,c, Bar plots comparing BioTranslator to existing methods in terms of unseen AUROC. We evaluated in Tabula Muris (Droplet), Tabula Microcebus and Tabula Sapiens. x-axis shows the proportion of unseen cell types in the test set. Error bar represents the standard errors in 5-fold cross-validation. Center of the error bar is the mean value.

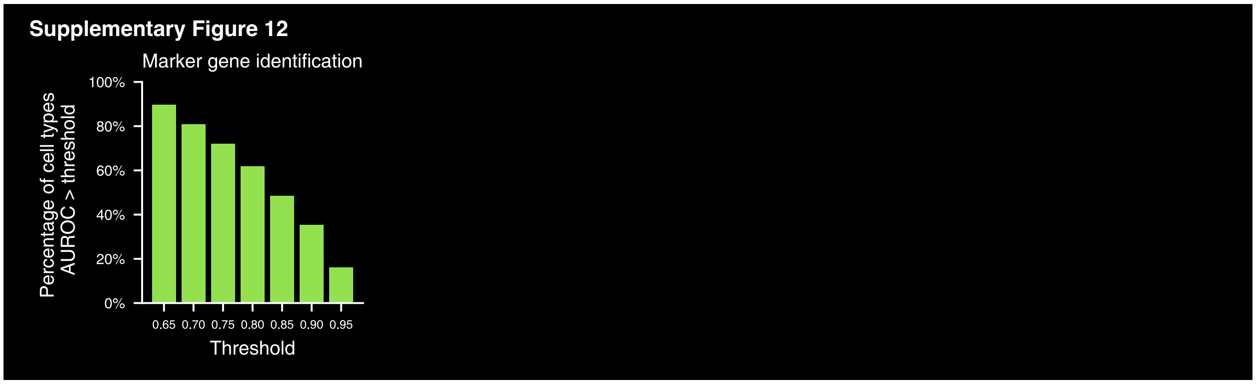

Supplementary Figure 12. Bar plot showing the AUROC performance of marker gene identification without using gene expression data. y-axis is the percentage of cell types with AUROC greater than different thresholds (x-axis).

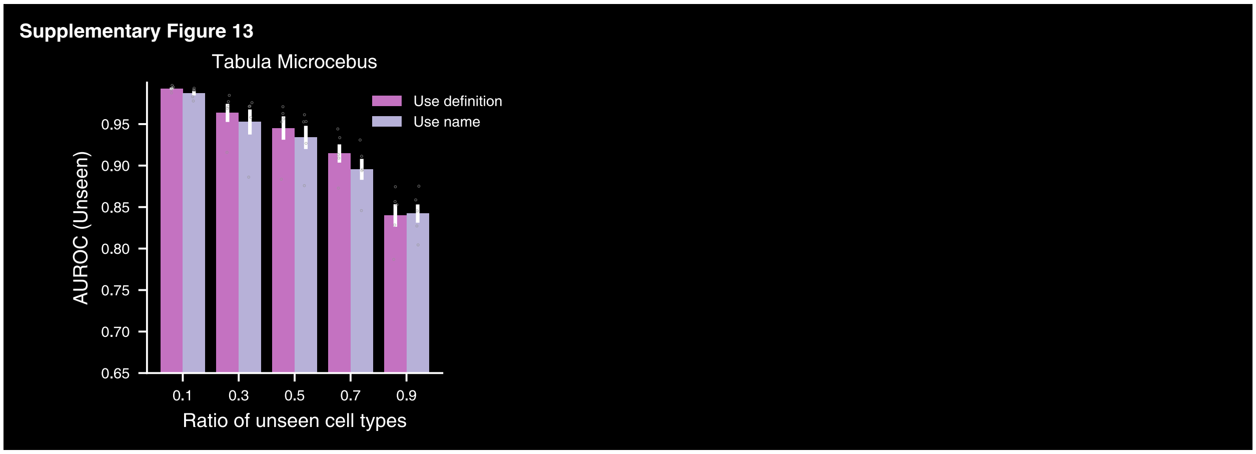

Supplementary Figure 13. Bar plot comparing the performance of BioTranslator using cell type definition to using cell type names on Tabula Microcebus. Definitions and names were obtained from the Cell Ontology. Error bar represents standard errors across 5-fold cross-validation and its center is mean value.
