# Supplementary material for: Multilingual translation for zero-shot biomedical classification using BioTranslator
Source: Nat Commun. 2023 Feb 10;14:738. doi: 10.1038/s41467-023-36476-2 (PMC9911740; doi:10.1038/s41467-023-36476-2)
Supplement: Supplementary file 3 — Reporting Summary [file 41467_2023_36476_MOESM3_ESM.pdf]

nature portfolio

Corresponding author(s): Sheng Wang

Last updated by author(s): Nov 13, 2022

Reporting Summary

Nature Portfolio wishes to improve the reproducibility of the work that we publish. This form provides structure for consistency and transparency in reporting. For further information on Nature Portfolio policies, see our Editorial Policies and the Editorial Policy Checklist.

Statistics

For all statistical analyses, confirm that the following items are present in the figure legend, table legend, main text, or Methods section.

n/a	Confirmed	
☐	☒	The exact sample size (*n*) for each experimental group/condition, given as a discrete number and unit of measurement
☐	☒	A statement on whether measurements were taken from distinct samples or whether the same sample was measured repeatedly
☐	☒	The statistical test(s) used AND whether they are one- or two-sided<br>*Only common tests should be described solely by name; describe more complex techniques in the Methods section.*
☒	☐	A description of all covariates tested
☒	☐	A description of any assumptions or corrections, such as tests of normality and adjustment for multiple comparisons
☐	☒	A full description of the statistical parameters including central tendency (e.g. means) or other basic estimates (e.g. regression coefficient) AND variation (e.g. standard deviation) or associated estimates of uncertainty (e.g. confidence intervals)
☒	☐	For null hypothesis testing, the test statistic (e.g. *F*, *t*, *r*) with confidence intervals, effect sizes, degrees of freedom and *P* value noted<br>*Give P values as exact values whenever suitable.*
☒	☐	For Bayesian analysis, information on the choice of priors and Markov chain Monte Carlo settings
☒	☐	For hierarchical and complex designs, identification of the appropriate level for tests and full reporting of outcomes
☒	☐	Estimates of effect sizes (e.g. Cohen's *d*, Pearson's *r*), indicating how they were calculated

Our web collection on statistics for biologists contains articles on many of the points above.

Software and code

Policy information about availability of computer code

Data collection	There is no software for data collection.
Data analysis	In the protein function prediction, we used scikit-learn Python package (v0.24.2) to calculate the AUROC values on the validation set. We used Mashup to calculate the network features for proteins. We used NLTK Python package (v3.7) to calculate the BLEU scores in generating textual description. In the single cell analysis section, we used scripts in OnClass (v1.9.1)(https://www.nature.com/articles/s41467-021-25725-x) to perform the single dataset cross-validation, cross-dataset validation and calculate the AUROC values of classification. We also used Cytoscape (v3.9.1) to plot the protein-protein interaction network, cell type marker gene network and the pathway network.

For manuscripts utilizing custom algorithms or software that are central to the research but not yet described in published literature, software must be made available to editors and reviewers. We strongly encourage code deposition in a community repository (e.g. GitHub). See the Nature Portfolio guidelines for submitting code & software for further information.

Data

Policy information about availability of data

All manuscripts must include a data availability statement. This statement should provide the following information, where applicable:

- Accession codes, unique identifiers, or web links for publicly available datasets
- A description of any restrictions on data availability
- For clinical datasets or third party data, please ensure that the statement adheres to our policy

The datasets used for protein function prediction and pathway analysis are available at: https://figshare.com/articles/dataset/Protein_Pathway_data_tar/20120447.

The processed datasets for cell type classification are available at: https://figshare.com/ndownloader/files/28846647 and https://figshare.com/ndownloader/files/31777475. Other datasets used for single cell analysis are obtained from OnClass. The gene to text association file is obtained from https://stringdb-static.org/download/protein.info.v11.5/9606.protein.info.v11.5.txt.gz, the drug to text association files is available at: https://raw.githubusercontent.com/blender-nlp/MolT5/main/ChEBI-20_data/train.txt, the phenotype to text file is available at: https://raw.githubusercontent.com/obophenotype/human-phenotype-ontology/master/hp.obo. The GDSC dataset and STITCH dataset can be found at: https://www.cancerrxgene.org/downloads/drug_data and http://stitch.embl.de/download/protein_chemical.links.detailed.v5.0/9606.protein_chemical.links.detailed.v5.0.tsv.gz. The gene to phenotype association file and pathway to phenotype association file are at: https://data.monarchinitiative.org/latest/tsv/all_associations/index.html.

Human research participants

Policy information about studies involving human research participants and Sex and Gender in Research.

Reporting on sex and gender	N.A.
Population characteristics	N.A.
Recruitment	N.A.
Ethics oversight	N.A.

Note that full information on the approval of the study protocol must also be provided in the manuscript.

Field-specific reporting

Please select the one below that is the best fit for your research. If you are not sure, read the appropriate sections before making your selection.

- [x] Life sciences
- [ ] Behavioural & social sciences
- [ ] Ecological, evolutionary & environmental sciences

For a reference copy of the document with all sections, see nature.com/documents/nr-reporting-summary-flat.pdf

Life sciences study design

All studies must disclose on these points even when the disclosure is negative.

Sample size	We determine the sample size by cross-validation. In the protein function prediction, we performed 3-fold cross validation, each fold was chosen randomly from the dataset. We chose 3-fold cross validation because the variances of evaluation performance were already sufficient small. In the single cell analysis, we used 5-fold cross validation. We chose 5-fold cross validation to keep consistent with the process in OnClass (v1.9.1).
Data exclusions	We fine-tuned PubMedBert using 225 ontologies and excluded the Gene Ontology and Cell Ontology to avoid the graph information leakage.
Replication	We used the 3-fold cross validation in protein function prediction and 5-fold cross validation to simulate the replication in single cell replication. When evaluating the baselines for classification between drug, gene, phenotype and pathway modalities, we used 5-fold cross validation. We confirm all attempts at replication were successful.
Randomization	We used Randomization in cross-validation. We randomly split data into different folds.
Blinding	The investigators were blinded to group allocation.

Reporting for specific materials, systems and methods

We require information from authors about some types of materials, experimental systems and methods used in many studies. Here, indicate whether each material, system or method listed is relevant to your study. If you are not sure if a list item applies to your research, read the appropriate section before selecting a response.

Materials & experimental systems

n/a	Involved in the study	
x		Antibodies
x		Eukaryotic cell lines
x		Palaeontology and archaeology
x		Animals and other organisms
x		Clinical data
x		Dual use research of concern

Methods

n/a	Involved in the study	
x		ChIP-seq
x		Flow cytometry
x		MRI-based neuroimaging
